# 2119. Predictive Value of the Immunodeficiency Scoring Index for COVID-19 Related Outcomes in Hematopoietic Transplant Recipients

**DOI:** 10.1093/ofid/ofac492.1740

**Published:** 2022-12-15

**Authors:** Marilyne Daher, Fareed Khawaja, Georgios Angelidakis, Gabriella Rondon, Amy Spallone, Jeremy Ramdial, Ella Ariza-Heredia, Elizabeth Shpall, Roy F Chemaly

**Affiliations:** Baylor College of Medicine, Houston, Texas; The University of Texas MD Anderson Cancer Center, Houston, Texas; The University of Texas Md Anderson Cancer Center, Houston, Texas; The University of Texas MD Anderson Cancer Center, Houston, Texas; University of Texas MD Anderson Cancer Center, Houston, Texas; University of Texas MD Anderson Cancer Center, Houston, Texas; The University of Texas MD Anderson Cancer Center, Houston, Texas; The University of Texas MD Anderson Cancer Center, Houston, Texas; MD Anderson, Houston, Texas

## Abstract

**Background:**

The Coronavirus Disease 2019 (COVID-19) has significantly impacted cancer patients with some reported mortality as high as 25%. The Immunodeficiency Scoring Index (ISI) was developed as a prognostic tool in allogeneic hematopoietic cell transplant (allo-HCT) recipients with respiratory syncytial virus but also for other respiratory viruses to predict severe infections and mortality. The purpose of our study was to correlate the ISI in HCT recipients with COVID-19 and associated complications such as hospitalization, supplemental oxygen use, and mortality.

**Methods:**

We performed a cohort study of HCT recipients of all ages with COVID-19 between March 2020 and October 2021. We included only patients who were diagnosed by a PCR-based assay. We excluded patients for whom an ISI score, as previously described, could not be calculated. Outcomes of interest included 60-day mortality, hospital and ICU admission due to COVID-19, and supplemental oxygen requirements. A univariate analysis using Fischer exact testing for nominal variables was performed.

**Results:**

Out of the 219 HCT with COVID-19, 101 were excluded due to alternative methods of diagnosis (13), lack of laboratory values needed to calculate an ISI at time of COVID-19 diagnosis (79), or COVID-19 diagnosed prior to transplant (9). Out of the remaining 118 patients, the median age was 60 years (range 6-85), most were male (56%), Caucasian (57%), and had no smoking history (64%). Most patients had an alloHCT (66%) with matched related donor [MRD] (25%), or matched unrelated donor [MUD] (21%) (Table 1). Median time from transplant to COVID-19 was 615 days (range 2-5692), median ISI was 3 (range 0-11), and 92% of patients were unvaccinated prior to COVID-19 (Table 1). On univariate analysis, an ISI of moderate to high (score ≥3) was associated with COVID-19 related hospitalization [p=0.0147] and an ISI ≥ 4 was associated with 60-day all-cause (p=0.045) and COVID-19-related (p-0.019) mortality (Table 2).

**Table 1:**
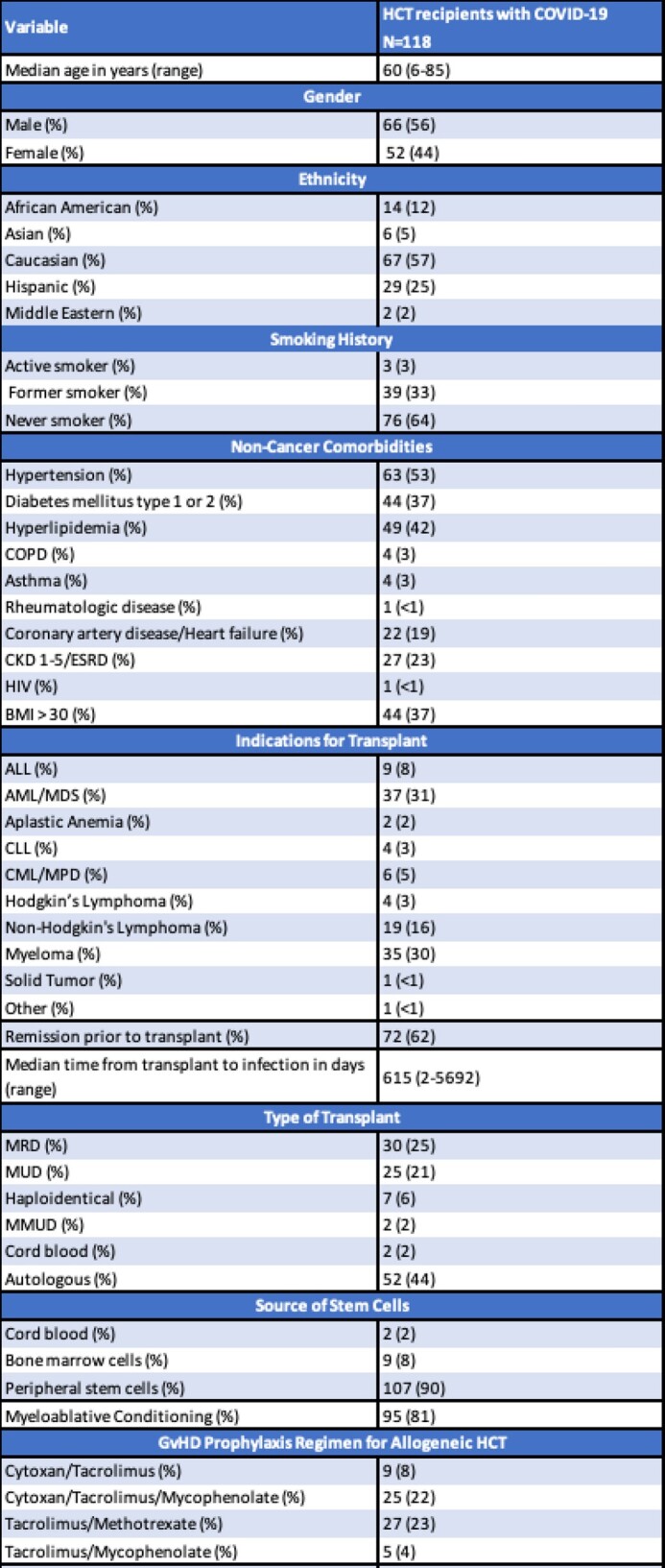
Patient Characteristics

**Table 2:**
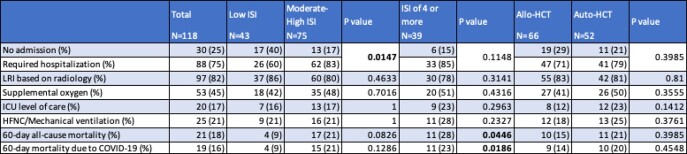
Univariate Analysis of Outcomes due to COVID-19 in HCT Recipients

**Figure 1:**
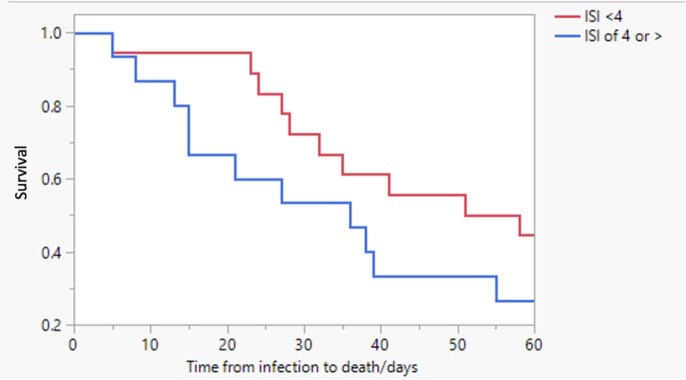
Survival Curve (Kaplan-Meier Curve) Comparing Time to Death in Patients with an ISI Score of 4 or Greater. (Log-rank 0.0295)

**Conclusion:**

An ISI of 4 or greater was a prognostic marker for worse outcomes such as COVID-related and all-cause mortality in HCT recipients. Whether an aggressive and prompt management of high-risk patients with COVID-19 may impact these outcomes needs to be determined in future studies.

**Disclosures:**

**Gabriella Rondon, MD**, Omeros: Advisor/Consultant **Ella Ariza-Heredia, MD**, MERCK: Grant/Research Support **Elizabeth Shpall, MD**, Adaptimmune: Advisor/Consultant|Affimed: License agreement|Axio: Advisor/Consultant|Bayer Helathcare Pharmaceuticals: Honoraria|Fibroblasts and FibrioBiologics: Advisor/Consultant|Navan: Advisor/Consultant|NY Blood Center: Advisor/Consultant|Takeda: License agreement **Roy F. Chemaly, MD/MPH**, Karius: Advisor/Consultant|Karius: Grant/Research Support.

**Table 1:** Patient Characteristics

Abbreviations: Hematopoietic Cell Transplant (HCT); Chronic Obstructive Pulmonary Disease (COPD); Chronic Kidney Disease (CKD); End-Stage Renal Disease (ESRD); Acute Lymphocytic Leukemia (ALL); Acute Myeloid Leukemia (AML); Myelodysplastic Syndrome (MDS); Chronic Lymphocytic Leukemia (CLL); Chronic Myelogenous Leukemia (CML) Myeloproliferative Disorder (MDS); Matched Related Donor (MRD); Matched Unrelated Donor (MUD); Mismatched Unrelated Donor (MMUD); Graft versus Host Disease (GvHD); Lower Respiratory Tract Infection (LRI); White Blood Cell (WBC); Absolute Neutrophil Count (ANC); Absolute Lymphocyte Count (ALC).

**Table 2:** Univariate Analysis of Outcomes due to COVID-19 in HCT Recipients

Abbreviations: Immunodeficiency Scoring Index (ISI); Hematopoietic Cell Transplant (HCT); Allogenic (Allo); Autologous (Auto); Lower Respiratory Infection (LRI); High-Flow Nasal Cannula (HFNC)

